# Standardizing Normal Reference Value for Thyroid Uptake of Technetium-99m Pertechnetate in Nepalese Population

**DOI:** 10.1055/s-0044-1779283

**Published:** 2024-02-13

**Authors:** Jiwan Paudel, Babita Bhattarai

**Affiliations:** 1 Department of Nuclear Medicine, Chitwan Medical College, Bharatpur, Nepal; 2 Nepal Army Institute of Health Sciences, Bhandarkhal, Saanobharyang, Nepal

**Keywords:** normal, pertechnetate, reference, thyroid, uptake

## Abstract

**Objective** Changes in normal reference values of thyroid uptake for iodine have been reported due to geographical and chronological fluctuations in dietary iodine intake in different populations. Nepal is a country with mixed ethnicity, with access to dietary iodine in the form of successful universal salt iodination program by the government of Nepal since 1973. The aim of this study was to establish the normal reference values for thyroid uptake of technetium-99m (Tc-99m) pertechnetate in the Nepalese population in iodine sufficiency era.

**Methods** We prospectively evaluated 52 clinically and biochemically euthyroid participants (46 females and 6 males) with age range from 20 to 71 years who underwent a thyroid Tc-99m pertechnetate scan and uptake between December 2019 to November 2023 in the Department of Nuclear Medicine, Chitwan Medical College fulfilling inclusion/exclusion criteria. Biochemical thyroid function tests were reviewed and Tc-99m pertechnetate thyroid uptake values were determined for each patient. Blood was withdrawn for thyroid hormone assessment. Euthyroid participants were then administered 3.5 to 4.5 mCi of Tc-99m pertechnetate intravenously and their percentage thyroid uptake was calculated after 20 minutes.

**Results** The mean and median uptake of Tc-99m pertechnetate in euthyroid patients were 1.26 and 0.85%, respectively, and the interquartile range was 0.7 to 1.7%. The normal reference uptake value for Tc-99m pertechnetate in the studied population ranged between 0.3 and 3.6%. The fifth and 95th percentiles for pertechnetate uptake were 0.5 and 2.9%, respectively.

**Conclusion** The normal reference range for Tc-99m pertechnetate thyroid uptake in Nepalese population was 0.5 to 2.9% that is lower than the currently accepted international standard of 0.75 to 4.5%. Uptake also increased with increasing age. This study highlights the importance of periodically redefining the geographic location specific normal thyroid uptake reference values.

## Introduction

Thyroid gland function can be evaluated in two ways. Laboratory assessment by measurement of serum-free triiodothyronine (T3), free tetraiodothyroxine (fT4), and thyroid-stimulating hormone (TSH) is an indirect way of assessing thyroid gland function. Thyroid uptake and scintigraphy performed in the Department of Nuclear Medicine is a direct way of assessing thyroid function. It has the advantage of evaluating the gland structure in addition to its function.

Radioiodine (I-131 Iodide) was first introduced in 1946. Its potential for the diagnosis and management of thyroid diseases and thyroid cancer was soon appreciated. It was the first radioisotope used for measuring thyroid uptake, and for several years it has remained the primary imaging agent for evaluating thyroid function. 1 Despite the high sensitivity and specificity of the currently used in vitro tests for the evaluation of thyroid function, thyroid uptake and scintigraphy play an important role in various clinical settings, such as differential diagnosis of thyrotoxicosis. When there is a strong clinical suspicion in a biochemically confirmed thyrotoxic patient, increased uptake in the thyroid gland is indicative of hyperthyroidism while decreased uptake suggests other clinical conditions such as subacute thyroiditis, Hashimoto's thyroiditis, extrathyroidal cause of thyrotoxicosis, thyrotoxicosis factitia, and drug interference. Thyroid uptake and scintigraphy also play an important role for therapeutic decision making in various clinical settings, such as functional assessment and characterization of nodule(s), identification of other causes of thyrotoxicosis, detection and localization of ectopic thyroid tissue, and calculation of therapeutic doses of I-131 iodide. 2

In a normal euthyroid healthy individual, technetium-99m (Tc-99m) pertechnetate or radioactive iodine uptake (RAIU) values depend upon iodine reserves in the thyroid gland that in turn varies primarily based on the long-term dietary intake of iodine. 3 4 Pertechnetate and iodine are both transported into the thyroid gland by the same sodium iodide symporter present in the thyroid follicular cells. The normal thyroid uptake reference values for Tc-99m pertechnetate and radioactive iodine in euthyroid person vary with the geographical location, and may change from one decade to the next because of change in dietary intake and supplementation of iodine fortified foods. Since there are geographical variations in the dietary iodine content, Tc-99m pertechnetate or RAIU values may also vary accordingly. 5 Hence, there is a need for re-defining the local Tc-99m pertechnetate or RAIU reference values periodically for accurate diagnosis of thyroid diseases as well as verification of laboratory reports.

Thyroid uptake values that are currently used in various nuclear medicine departments across the globe were established since the 1960s when there was widespread iodine deficiency across various geographic regions. With the introduction of Universal Salt Iodination Program from Nepal government since 1973, the prevalence of iodine deficiency has decreased significantly. In the current setting of iodine sufficiency in Nepal, the normal pertechnetate or RAIU values are expected to be lower than those international references established earlier. With the current pertechnetate or RAIU reference values, many patients with upper normal uptake values may be misdiagnosed as being normal. Therefore, there is a need to re-establish the pertechnetate or RAIU local reference range in the iodine sufficiency era.

A number of radioisotopes have been used for thyroid uptake studies. I-131 iodide has the disadvantage of high radiation doses delivered to the gland (1–3 rad/mCi) because of its long half-life of 8.2 days and 606 keV beta *-* particle emission. Its high principal gamma photon energy of 364 keV is inadequately collimated by the commonly used scintillation gamma cameras, and therefore they produce poor quality images. Thus, I-131 iodide for thyroid imaging has been restricted only to staging and follow-up of patients with differentiated thyroid cancer. 2 6 Even in developing countries like Nepal, it is not used for imaging of thyroid gland.

I-123 is preferable to I-131 and is the imaging agent of choice as of 2023 because it has a shorter half-life of 13 hours and 159 keV gamma photon suitable for imaging with current scintillation cameras. It is devoid of beta *-* radiation; thus, it has a favorable radiation dosimetry. However, it is not available in developing countries like Nepal because it is cyclotron produced and is expensive. Contaminants such as I-124 iodide and I-125 iodide are frequently encountered increasing the radiation dosimetry and degrading the image quality. 7 8 Currently Tc-99m in the form of pertechnetate ( ^99m^ TcO _4_ -) is the most commonly used agent for thyroid scintigraphy and uptake measurement using scintillation gamma cameras in developing countries. The similarity of volume and charge between the iodide and pertechnetate ions explains that Tc-99m pertechnetate is taken up by the thyroid gland by the same sodium iodine symporter that uptakes iodine. 9 10 It is trapped by the thyroid follicular cells by the same mechanism as iodine but unlike iodine it is neither organified nor incorporated into thyroid hormones.

Tc-99m pertechnetate is extensively used across the globe because of a number of advantages: (1) short half-life of 6 hours, (2) short retention in the gland, (3) devoid of beta *-* radiation, (4) favorable radiation dosimetry to the thyroid gland (10,000 times less than that of I-131) and to the whole body, (5) its principal gamma photon of 140 keV is ideal for collimation using scintillation cameras, and (6) it is generator produced thus inexpensive, and (7) readily available form Molybdenum-Technetium generator. 11 Although Tc-99m pertechnetate is only trapped but not organified by the thyroid gland, in the majority of cases the uptake and imaging data provide all necessary information for accurate clinical diagnosis. 12 In rare instances, where available, I-123 can subsequently be used for the assessment of organification defects.

In Nepal, there are mixed ethnic population with universal access to dietary iodine in the form of iodized salt since 1973. Despite historical reports on deviating normal thyroid uptake values in different geographical location (thus highlighting the importance of establishing local standard normal reference values), the concerned Nepalese authorities have neither revised nor established these reference values. The reason for this is that only a few centers are available for nuclear thyroid scintigraphy and uptake studies in Nepal as of 2023. Since Chitwan Medical College (CMC) lies in the central region of Nepal and given the setting of only a few nuclear medicine centers in the entire country, patients visiting CMC are ideal representatives of all ethnic groups including Brahmins, Newars, Janajatis, and Chhetris and all geographical location including terai, hills, mountains, and Himalayas all over Nepal. The aim of this study was to standardize and establish the normal reference values for thyroid uptake of Tc-99m pertechnetate in the Nepalese population.

## Materials and Methods

We prospectively evaluated 52 normal euthyroid individuals comprising 46 females and 6 males (female-to-male ratio 7.6:1 and mean age of 38.6 ± 12.0 years within age range of 20 to 71 years) in the Department of Nuclear Medicine, CMC, Bharatpur, Nepal during the period from December 2020 to November 2023.

Prior laboratory assessment of recent thyroid function tests was obtained via serum measurements of free tetraiodothyroxine (fT4) and TSH that were all within normal limits of the institution's laboratory range. Quantitative determination of the serum TSH was assessed in vitro using ADVIA Centaur, ADVIA Centaur XP, and ADVIA Centaur XPT systems TSH assay using two-site sandwich immunoassay with direct chemiluminometric technology, which uses constant amounts of two antibodies. The first antibody, in the Lite Reagent, is a monoclonal mouse anti-TSH antibody labeled with acridinium ester. The second antibody, in the Solid Phase, is a polyclonal sheep anti-TSH antibody that is covalently coupled to paramagnetic particles. Quantitative determination of free thyroxine (FT4) in serum or plasma (heparinized or ethylenamineintetraacetic acid) was also performed using the ADVIA Centaur, ADVIA Centaur XP, and ADVIA Centaur XPT systems that are competitive immunoassay using direct chemiluminescent technology. The study protocol was approved by the Ethics Committee of CMC, Tribhuvan University. All individuals were on a low iodine diet 2 weeks prior to the thyroid uptake and scintigraphy study. Clinically euthyroid individuals aged more than 18 years with no evidence of nodules or enlarged gland on palpation along with normal scan and background findings confirmed by scintigraphic images were selected for the study.

Each subject was selected by using a questionnaire that evaluated the clinical history, history of iodinated-contrast radiographic procedures, and a physical examination to exclude those with thyroid, cardiac, or renal diseases. Individuals with iodine contamination such as use of iodex for local application were also excluded. Participants taking medications known to affect thyroid function such as thyroid hormones, antithyroid medications like methimazole, carbimazole, or propylthiouracil, amiodarone, Lithium, cough syrups, multivitamins, any ayurvedic, homeopathic and desi medicines or patients with recent ingestion of sea foods were excluded from the study. Patients with a history of radionuclide administration within 6 months of thyroid scanning, previous thyroid surgery or radioiodine treatment and pregnant and lactating women were also excluded in the study.

**Fig. 1:**
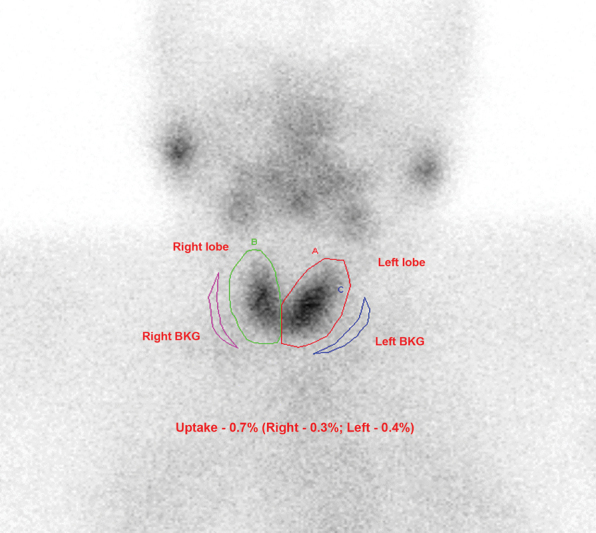
Normal uptake of technetium-99m pertechnetate (0.7%: right—0.3%; left—0.4%).

Each participant received 3.5 to 4.5 mCi of Tc-99m pertechnetate intravenously. The percentage of Tc-99m pertechnetate taken up by the thyroid gland was determined at 20 minutes, using scintigraphic imaging techniques with the Siemens Intevo Bold Hybrid single-photon emission tomography/computed tomography with a dual head digital gamma camera equipped with a low energy, high-resolution parallel hole collimator. First Tc-99m pertechnetate dose was measured in the Cap-In-Tec dose calibrator. Then radioactivity counts in the syringe before and immediately after injection were obtained for 15 seconds each for uptake calculation and the exact time of injection was noted in all cases. A 20% energy window was centered at 140 keV. All patients were asked to drink water 10 minutes before imaging to clear any Tc-99m pertechnetate labelled salivary activity from the esophagus. Anterior spot view mages of the neck were obtained at 20 minutes of injection at a preset data of 750k counts for studying the structure and shape of the thyroid gland ( Fig. 1 ). The neck was slightly extended with a support at the back during the scanning time and the camera head placed close over the neck with a matrix size of 128 × 128 and at zoom of 2. A control image of the radiopharmaceutical injection site was also obtained to confirm that there was no subcutaneous extravasation of the injected dose that would otherwise invalidate the uptake percentage calculation. The method for calculation of thyroid uptake, based on images of the gland obtained and syringe counts before and after radiopharmaceutical injection, was previously described by Maisey et al 13 and simplified for routine use. 14 The number of counts present in the thyroid (T) was determined by manually drawing the region of interest (ROI) along the borders of the right and left lobes of the gland ( Fig. 1 ). Other two ROIs were again drawn manually adjoining to the right and left lobes for background subtraction (BG) ( Fig. 1 ). The counts in the syringe before (B, i.e., presyringe counts) and after (A, i.e., postsyringe counts) radioisotope injection were obtained directly from the images. All counts were corrected for the acquisition time and decay of Tc-99m by the computer. The thyroid uptake (TU) was calculated from the equation: TU = (T–BG)/(B–A).

## Results

Thyroid uptake of Tc-99m pertechnetate in the study population ranged from 0.3 to 3.6%. Mean uptake was 1.26%, median uptake was 0.85%, and interquartile range was 0.7 to 1.7%. The mean (± standard deviation) Tc-99m pertechnetate uptake for males and females were 1.1 ± 0.7 and 1.28 ± 0.79%, respectively, and overall mean pertechnetate uptake in the population was 1.26 ± 0.78% ( Tables 1 and 2 ).

**Table 1:** TSH values and Tc-99m pertechnetate uptake in normal population

		Age Median (IQR)	TSH Median (IQR)	Uptake Median (IQR)
Overall		37.5 (28.0–47.0)	2.28 (1.19–4.20)	0.85 (0.7–1.7)
Gender	Male	39.0 (38.0–56.5)	2.0 (0.92–2.05)	0.75 (0.57–2.00)
	Female	35.5 (27.75–47.0)	2.4 (1.19–4.31)	1.0 (0.7–1.7)

Abbreviations: IQR, interquartile range; Tc-99m, technetium-99m; TSH, thyroid-stimulating hormone.

**Table 2:** Mean and ranges of uptake in normal individuals

Group	Total	%	Mean age (y)	Age range (y)	% uptake range	% uptake mean ± SD	% uptake 95% CI
Male	6	11.5	44.67	38–58	0.5–2.0	1.1 ± 0.70	0.36–1.84
Female	46	88.5	37.87	20–71	0.3–3.6	1.28 ± 0.79	1.04–1.52
Population	52	52	38.65	20–71	0.3–3.6	1.26 ± 0.78	1.04–1.48

Abbreviations: CI, confidence interval; SD, standard deviation.

On comparing the gender-specific data, gender did not seem to play a significant role in euthyroid pertechnetate uptake, but normal values appeared to be marginally lower in men (1.1 ± 0.7%) than in women (1.3 ± 0.8%) ( Table 2 ).

**Fig. 6:**
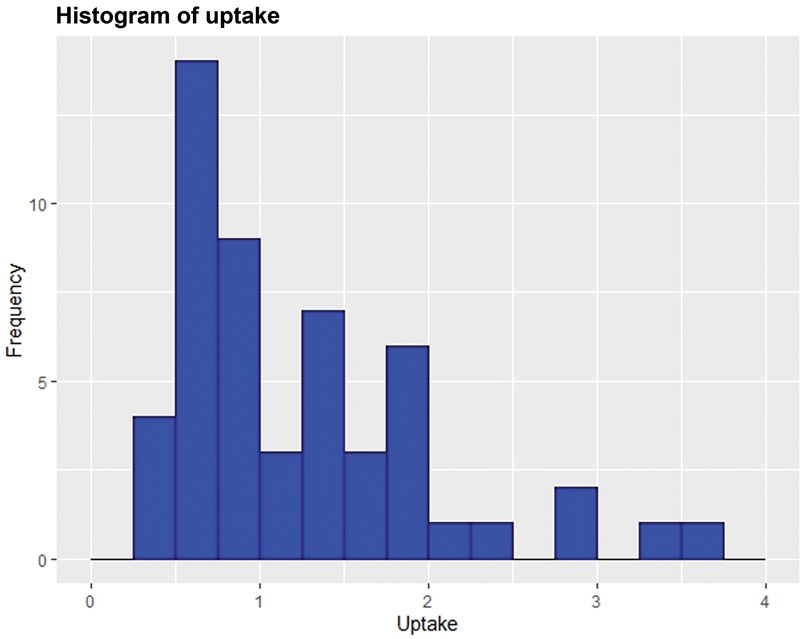
Frequency histogram of mean uptake of technetium-99m pertechnetate in Nepalese population.

**Fig. 7:**
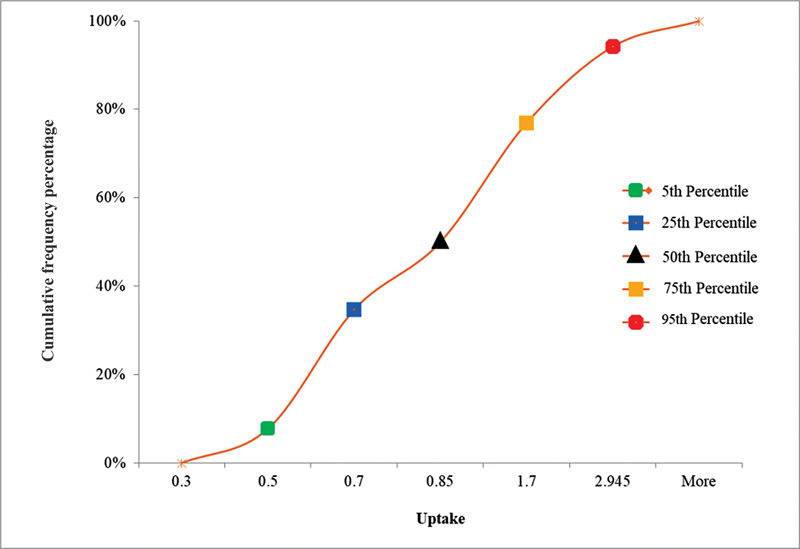
Cumulative frequency polygon with percentile of mean technetium-99m pertechnetate uptake in Nepalese population.

To determine if the uptake was distributed normally in the Nepalese population, uptake values were grouped over intervals of 0.25% (0–0.25, > 0.25–0.50, > 0.50–0.75, and so on) and a histogram of the mean uptake for the intervals generated ( Fig. 6 ). These data clearly showed that pertechnetate uptake was skewed and not normally distributed. Eight percent of the participants ( *n* = 4) showed extremely low pertechnetate uptake (≤ 0.5%). Thirty-five percent of the participants ( *n* = 18) had uptake values below the departmental lower limit of 0.75%. The remaining 57% of participants had uptake values less than or equal to 3.6% (within the international and departmental reference range of 0.75–4.5%). Because of skewness in uptake distribution, we determined the reference range for Tc-99m pertechnetate by first plotting a cumulative frequency curve and determined 5th and 95th percentiles to represent the normal reference range for the euthyroid Nepalese population. The 5th and 95th percentiles were in the range of 0.5 to 2.9% ( Fig. 7 ).

## Discussion

The existing literature on thyroid scintigraphy and uptake shows a number of radiotracers currently used across the globe depending on the availability and indications of the scan and uptake. Tc-99m pertechnetate is increasingly used as the radiotracer of choice in patients with thyrotoxicosis because the result of the uptake is obtained in one visit 15 16 in less than an hour time. Currently I-131 is not used for routine thyroid scintigraphy because of its high radiation dosimetry and poor image quality. Its use is thus restricted to extremely low doses (< 30 μCi) for obtaining uptake values for high-dose therapies for differentiated thyroid cancer and hyperthyroidism from various causes. Currently used radioisotopes for thyroid scintigraphy and uptake are I-123 iodide and Tc-99m pertechnetate. However, I-123 iodide, being cyclotron produced, is expensive and not available in developing countries like Nepal. Therefore, Tc-99m pertechnetate has become the tracer of choice because it is readily available from Mo-Tc generator that can be easily shipped to regional radiopharmacies. The use of I-123 iodide is restricted to studies that require iodide organification such as organification defects in autoimmune chronic thyroiditis and congenital dyshormonogenetic hypothyroidism. 9 10

The maximum thyroid uptake of Tc-99m pertechnetate takes place at approximately 20 minutes after intravenous injection of radioisotope, in contrast to I-131 iodide, which requires a 24-hour uptake measurement period. Tc-99m pertechnetate uptake by the thyroid gland is low and the currently used reference range by most of the laboratories in the world is 0.75 to 4.5%. 17 18 But lower normal reference values have been described in different populations in various literature. Studies have shown that normal values of Tc-99m pertechnetate uptake depend on the technique used and on the dietary intake of iodide. Thus, each laboratory should establish its own normal reference values for its local population.

**Fig. 4:**
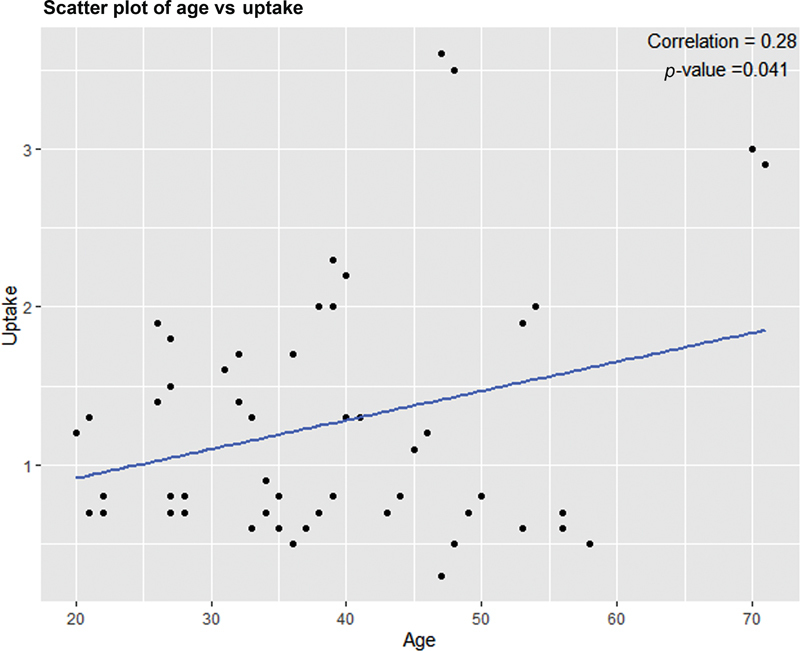
Relationship between technetium-99m pertechnetate and age (total euthyroid population).

This study assessed the normal values for thyroid Tc-99m pertechnetate uptake in Nepalese population who were clinically and biochemically euthyroid. The normal reference range for Tc-99m pertechnetate uptake used by our institute, a few other centers in Nepal and many others across the globe (0.75–4.5%), is relatively wide. In our study, the normal Tc-99m pertechnetate uptake reference range was 0.5 to 2.9%. This range of thyroid uptake of Tc-99m pertechnetate in our study differed significantly from the currently used international accepted reference range. Both the upper and the lower range limits are lower than the international accepted standard range. These values are similar to other literature data from other regions outside Nepal. Normal reference ranges for thyroid uptake of Tc-99m pertechnetate vary significantly in different geographic regions. 19 20 Recently published geographic specific literature data are as follows: Macauleya et al (0.5–1.4%) from the UK, Ramos et al (0.4–1.7%) from Brazil, and Hamunyela et al (0.15–1.69%) from Namibia. 19 21 22 All of these studies show normal reference values significantly lower than those currently used in most of the nuclear medicine laboratories. Our study also closely matches these lower limits of normal reference values proposed by different investigators in different countries across the globe. However, upper limit of normal was slightly higher in our study, that is, 2.9%. This variation is likely attributed to geographic variation and dietary content of iodine in iodine fortified foods in this part of the world. To our knowledge, there are no published reports of normal reference values for thyroid Tc-99m pertechnetate uptake from institutions in Nepal or South Asian countries. This study aimed to evaluate the normal values of Tc-99m pertechnetate in the Nepalese population that will likely represent south Asian population too. However, in contrast to study by Hamunyela et al that revealed decreased uptake with increasing age, our study showed Tc-99m pertechnetate uptake increases with increasing age of the individuals and the increased uptake with age was statistically significant ( *p* = 0.04; Fig. 4 ). 21

The uptake values for 52 individuals revealed a non-Gaussian distribution, as previously observed by Maisey et al. 13 In this study, the range of euthyroid uptake derived from Tc-99m pertechnetate was significantly lower (0.5–2.9%) than the internationally accepted normal reference values (0.75–4.5%) that are currently used by our institute (Department of Nuclear Medicine, CMC, Nepal). The data in Fig. 6 demonstrate that more than 50% of participants had very low uptake values below 1.0%. On the basis of the departmental reference values, 35% ( *n* = 18) of these participants would have been diagnosed as abnormal. There is considerable overlapping and significant differences between these data with those reported by other investigators.

Thyroid uptake of radioiodine has shown persistent decline in the past decades. Lowering of normal uptake values has been reported in Indian population that has ethnic and geographic similarities with Nepalese population. 23 Some investigators have suggested that medication that contains supplements may be responsible for the lowering of normal values of iodine. Anderson and Powsner demonstrated that an increase in iodine ingestion in iodine fortified food was a major cause of decreased reference values. 24

Our study suggested that gender did not appear to significantly affect uptake of Tc-99m pertechnetate ( Table 2 ). The reasons for the low pertechnetate uptake values in our study may be attributed to diet and dietary supplementation of iodine in the form of successful universal salt iodination program by Nepal government since 1973. The continuous intake of iodized salt could have reduced thyroid uptake of Tc-99m pertechnetate because thyroid uptake of iodine directly correlates with Tc-99m pertechnetate uptake since the mechanism of uptake of the two radioisotopes by the thyroid is similar. Reinhardt et al demonstrated that thyroid uptake of pertechnetate inversely correlated with iodine intake. 25 Thus, thyroid uptake of pertechnetate tends to be low in iodine sufficient population as in our study.

## Conclusion

We found a marked lowering of normal reference range for Tc-99m pertechnetate thyroid uptake in Nepalese population. The normal reference range of pertechnetate uptake in Nepalese population was 0.5 to 2.9% that is lower than the currently accepted international standard of 0.75 to 4.5%. Uptake also showed increasing trend with increasing age. These results highlight the importance of periodic evaluation and re-establishment of normal thyroid uptake reference values of Tc-99m pertechnetate in Nepalese population. Further studies are warranted to establish the age-adjusted geographic region-specific uptake values for the thyroid Tc-99m pertechnetate scan.

**Fig. 2:**
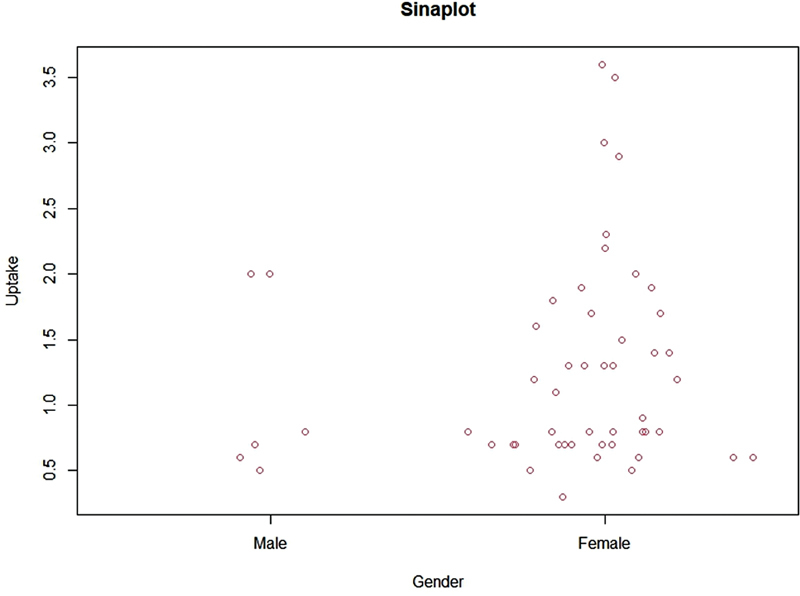
Technetium-99m pertechnetate uptake by sex ( *n* = 52).

**Fig. 3:**
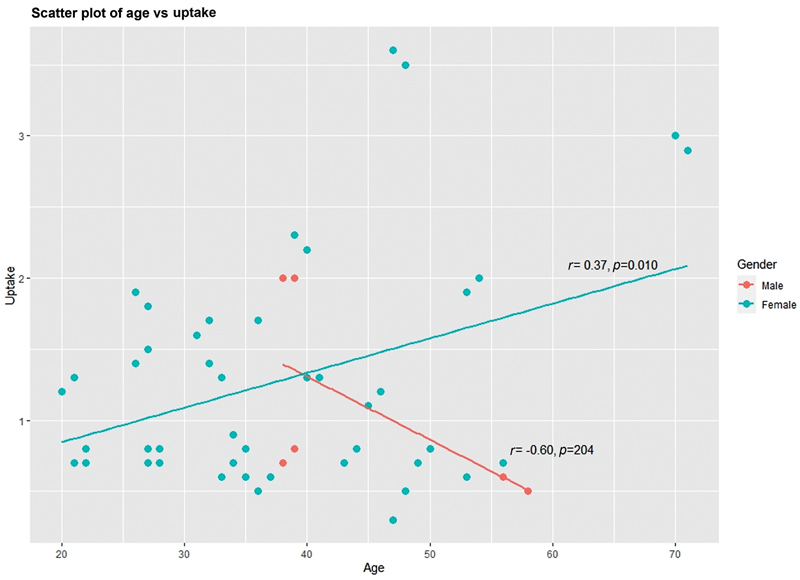
Relationship between technetium-99m pertechnetate uptake and age (males and females separate).

**Fig. 5:**
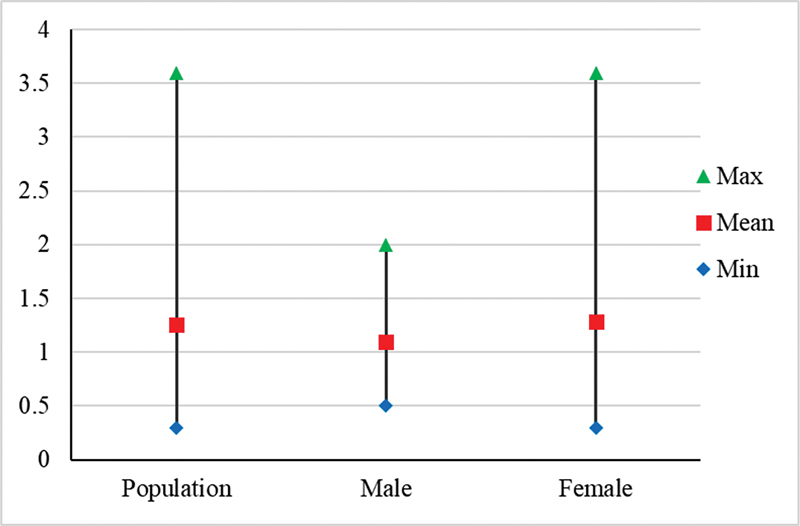
Gender comparison of technetium-99m pertechnetate uptake in euthyroid population.
